# Update on Nicotinamide and Its Application in the Management of Glaucoma

**DOI:** 10.3390/ijms262110789

**Published:** 2025-11-06

**Authors:** Ta-Hung Chiu, Shih-Heng Hung, Chiao-Hsin Lan, Wei-Ting Yen, Da-Wen Lu

**Affiliations:** 1 Department of General Medicine, Tri-Service General Hospital, National Defense Medical University, Taipei 114202, Taiwan; 2 Department of Ophthalmology, Show Chwan Memorial Hospital, Changhua 500, Taiwan; 3 Department of Medical Education, National Taiwan University Hospital, Taipei 100225, Taiwan; 4 Department of Ophthalmology, College of Medicine, National Defense Medical University, Taipei 114202, Taiwan; 5 Department of Ophthalmology, Tri-Service General Hospital, National Defense Medical University, Taipei 114202, Taiwan

**Keywords:** glaucoma, nicotinamide, vitamin B3, NAD^+^ metabolism, retinal ganglion cells, neuroprotection, salvage pathway

## Abstract

Glaucoma continues to be a primary contributor to permanent vision loss worldwide, frequently advancing even when intraocular pressure management is clinically adequate. Accumulating research emphasizes the metabolic susceptibility of retinal ganglion cells (RGCs), specifically concerning the progressive depletion of nicotinamide adenine dinucleotide (NAD^+^), a pivotal coenzyme fundamental to mitochondrial energy production and cellular survival mechanisms. As a key biosynthetic precursor in NAD^+^ synthesis pathways, nicotinamide (NAM), a form of vitamin B3, has exhibited significant neuroprotective properties across various preclinical experimental models and preliminary clinical investigations, demonstrating enhanced preservation of RGC morphology and physiological function. This comprehensive review systematically examines the current body of evidence supporting NAM’s therapeutic utility in glaucomatous neuroprotection, focusing particularly on underlying metabolic pathways, obstacles in clinical translation, and prospective therapeutic applications. Through systematic integration of data from cellular and molecular research, animal experimental studies, and population-based epidemiological investigations, we establish a conceptual framework for repurposing NAM as an innovative complementary therapeutic strategy in comprehensive glaucoma care, addressing key considerations for future clinical development including optimal dosing strategies, delivery mechanisms, and patient selection criteria for maximizing therapeutic outcomes in this challenging neurodegenerative condition.

## 1. Introduction

With more than 70 million people affected worldwide, glaucoma represents the leading etiology of irreversible visual impairment [1]. While lowering intraocular pressure (IOP) constitutes the primary therapeutic approach, numerous patients exhibit continued deterioration of visual function despite adequate pressure control, suggesting that pathophysiological processes independent of IOP contribute significantly to disease advancement [1]. This recognition has stimulated investigation into alternative neuroprotective interventions that target the fundamental cellular mechanisms underlying retinal ganglion cell (RGC) deterioration [1,2].

RGCs function as metabolically demanding neurons requiring optimal mitochondrial performance and nicotinamide adenine dinucleotide (NAD^+^)-mediated enzymatic processes for cellular survival [2]. Perturbations in NAD^+^ metabolic pathways—resulting from aging processes, environmental stressors, or pathological conditions—may precipitate bioenergetic dysfunction, elevated oxidative damage, and subsequent neuronal loss [3]. Within the spectrum of therapeutic approaches aimed at enhancing metabolic stability, administration of nicotinamide (NAM), a form of vitamin B3, serving as a biosynthetic substrate for NAD^+^ production, has demonstrated considerable therapeutic potential [4].

The present review investigates NAM’s possible application as supplementary treatment in glaucoma care, focusing on its diverse neuroprotective properties mediated through NAD^+^ metabolic restoration. We initially describe NAD^+^’s fundamental importance in RGC bioenergetics and examine the critical functions of salvage pathway components—specifically nicotinamide phosphoribosyltransferase (NAMPT), nicotinamide mononucleotide adenylyltransferase 1 (NMNAT1), and NMNAT2—in preserving mitochondrial integrity. Both experimental and clinical evidence are analyzed to demonstrate therapeutic potential alongside existing translational challenges, while outlining future investigational approaches to facilitate clinical implementation.

Recent publications have also reviewed the therapeutic implications of NAM in glaucoma [5,6]. Bhartiya (2022) [5] provided an editorial perspective highlighting NAM’s translational potential from bench to clinic, emphasizing its safety, accessibility, and neuroprotective promise. Babighian et al. (2024) [6] offered a comprehensive review of NAM’s cellular mechanisms, preclinical and clinical findings, safety profile, and comparison with other NAD^+^ precursors. Building upon these works, the present review provides an updated and integrative synthesis encompassing mechanistic, preclinical, and clinical evidence, while further addressing biomarker development, NAD^+^ salvage pathway regulation, and ongoing large-scale clinical trials. By integrating these recent advances and their translational implications, this review clarifies the evolving concept of glaucoma as a metabolic neurodegenerative disease and delineates NAM’s role within this emerging therapeutic framework.

## 2. Biological Role of NAD^+^ and NAM in RGC Metabolism

### 2.1. NAD^+^ as a Metabolic Hub in RGC Function

RGCs are uniquely energy-demanding neurons due to their long unmyelinated axons and reliance on oxidative phosphorylation (OXPHOS) for signal transmission [7,8]. Within these cells, NAD^+^ serves as a pivotal coenzyme linking mitochondrial metabolism, redox balance, and cell survival [9]. It is crucial for both adenosine triphosphate (ATP) generation through the tricarboxylic acid (TCA) cycle and electron transport chain (ETC), and as a cofactor for enzymes like sirtuins and poly (ADP-ribose) polymerases (PARPs) that mediate deoxyribonucleic acid (DNA) repair, cell signaling, and stress responses [10,11]. Given the high metabolic activity of RGC dendrites in the inner plexiform layer, disruptions in NAD^+^ homeostasis can trigger pseudohypoxia and compromise OXPHOS efficiency [11].

Recent human studies have confirmed that systemic NAD^+^ levels are positively correlated with mitochondrial oxygen consumption rate (OCR) in peripheral blood mononuclear cells (PBMCs), and that lower NAD^+^ is significantly associated with impaired mitochondrial respiration in glaucoma patients, particularly those with normal-tension glaucoma. These findings support the hypothesis that NAD^+^ availability directly modulates mitochondrial respiratory function, reinforcing the central role of NAD^+^ in maintaining RGC energy homeostasis [12].

### 2.2. NAD^+^ Salvage Pathway Enzymes in the Retina

**Figure 1:**
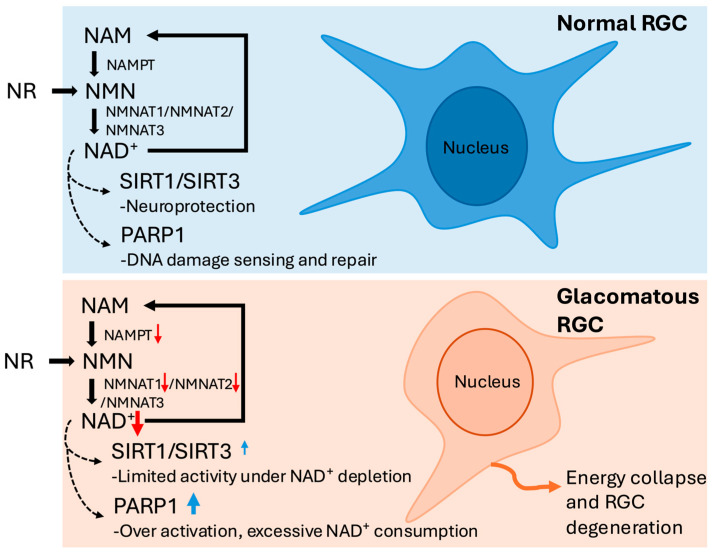
Schematic representation of the nicotinamide adenine dinucleotide (NAD^+^) salvage pathway in retinal ganglion cells (RGCs) under normal and glaucomatous conditions. Under normal conditions, nicotinamide (NAM) and nicotinamide riboside (NR) are converted to NAD^+^ through the sequential actions of nicotinamide phosphoribosyltransferase (NAMPT) and nicotinamide mononucleotide adenylyltransferases (NMNAT1–3). Adequate NAD^+^ levels sustain sirtuin 1/3 (SIRT1/SIRT3) activity for neuroprotection and support poly (ADP-ribose) polymerase 1 (PARP1) in physiological DNA repair. In glaucomatous RGCs, downregulation of NAMPT and NMNAT1/2, together with PARP1 overactivation, leads to NAD^+^ depletion and energy collapse. Although SIRT1/SIRT3 expression is increased, this reflects a compensatory response with limited activity under NAD^+^ deficiency, ultimately contributing to mitochondrial dysfunction and RGC degeneration. Red ↓ indicates decreased expression or activity; blue ↑ indicates compensatory or increased expression.

Building upon the understanding of NAD^+^’s essential metabolic role, attention must be turned to the mechanisms by which NAD^+^ is synthesized and maintained in the retina. In adult retinal tissue, the salvage pathway constitutes the predominant means of sustaining intracellular NAD^+^ levels, recycling NAM via the enzymes NAMPT and NMNATs [3] (Figure 1). In human RGCs, NAMPT catalyzes the formation of nicotinamide mononucleotide (NMN), followed by conversion to NAD^+^ through NMNAT isoforms [3]. NAMPT serves as the rate-limiting enzyme in this pathway and plays a critical role in determining intracellular NAD^+^ availability [13]. In RGCs, NMNAT1 predominantly localized to the nucleus, while NMNAT2 is cytosolic and enriched in axons, making them well-suited for supporting NAD^+^ biosynthesis in both somal and axonal compartments [14,15,16]. NMNAT2, specifically, is highly expressed in RGC axons and is crucial for axon maintenance [17]. Its expression declines with age and glaucoma severity, rendering the cells more susceptible to degeneration [2].

In late-stage glaucoma, NAMPT, NMNAT1, and NMNAT2 are downregulated in the ganglion cell complex (GCC) and optic nerve head (ONH), potentially impairing RGCs’ ability to regenerate NAD^+^; coupled with reduced plasma NAM, this suggests further limitation in NAD^+^ biosynthetic capacity [13,18].

### 2.3. NAD^+^ Metabolic Biomarkers as Translational Tools in Glaucoma

Beyond the impaired synthesis of NAD^+^, its excessive consumption by downstream enzymes also significantly influences RGC metabolic stability. The sirtuin (SIRT) family, particularly SIRT1 and SIRT3, are neuroprotective and upregulated in glaucomatous retinas [19,20]. SIRT1 suppresses oxidative stress and preserves mitochondrial integrity, yet its function is diminished under NAD^+^ depletion [19]. In contrast, PARP1 hyperactivation during oxidative DNA damage consumes large amounts of NAD^+^, leading to energy crisis and a form of cell death known as parthanatos [20]. In primary open angle glaucoma (POAG) patients, increased PARP1 concentrations have been detected in the aqueous humor, implicating overactivation of this enzyme—and subsequent NAD^+^ depletion—as a contributor to neurodegenerative processes [21].

Collectively, this evidence demonstrates that glaucoma extends beyond elevated IOP, representing a complex neurodegenerative disorder with profound metabolic vulnerability in RGCs. Declining NAD^+^ levels from impaired salvage enzymes and excessive PARP1 consumption create destructive cycles of mitochondrial dysfunction. Translational opportunities exist through NAD^+^ metabolic biomarkers—including systemic concentrations, retinal NAMPT/NMNAT expression, and PARP1 activity—for detecting metabolic distress, monitoring progression, and optimizing NAM-based therapeutic strategies.

## 3. Mechanistic Evidence: Animal Models and Cellular Studies

Experimental evidence from both murine and rat models has offered extensive insight into how NAD^+^ metabolism intersects with RGC vulnerability in glaucomatous degeneration [22,23].

These models have proven invaluable in dissecting time-dependent molecular cascades, enabling researchers to characterize not only when cellular dysfunction begins but also which pathways are modifiable through therapeutic intervention.

### 3.1. Early NAD^+^ Depletion and Mitochondrial Vulnerability in RGCs

In the DBA/2J mouse model of age-related chronic glaucoma, early mitochondrial dysfunction and reductions in NAD^+^ levels have been observed prior to any detectable structural damage [24]. Transcriptomic and metabolomic profiling reveal that age-related declines in NAD^+^ render RGC mitochondria more vulnerable to IOP–related stress, leading to early mitochondrial dysfunction, oxidative stress responses, and shifts in energy metabolism that predispose RGCs to degeneration [2,25]. These stressors include upregulation of eukaryotic Initiation Factor 2 (eIF2) and mammalian target of rapamycin (mTOR) signaling, mitochondrial fission, and DNA damage, accompanied by a decrease in both NAD^+^ and glutathione levels in RGCs even in the absence of overt cell loss [2].

The early onset of these metabolic disturbances suggests that RGCs exist in a “pre-degenerative” state that is amenable to rescue before cell loss becomes irreversible [2,22,26]. This state is marked by subtle shifts in transcriptomic signatures and bioenergetic homeostasis rather than overt cell death, thereby creating a critical therapeutic window [26]. Notably, the NAD^+^ salvage pathway is particularly compromised in this context, implicating enzymes such as NAMPT and NMNATs as central regulatory points of mitochondrial integrity [26].

### 3.2. NAM Supplementation Prevents RGC Dysfunction and Structural Loss

Building upon this evidence of early metabolic vulnerability, animal studies have demonstrated that NAM represents a potent therapeutic intervention. Oral NAM is the most extensively studied NAD^+^ precursor in glaucomatous models, with compelling evidence demonstrating that high-dose administration (e.g., 2000 mg/kg/day) elevates retinal NAD^+^ levels, prevents RGC soma and axon loss, preserves axoplasmic transport, and sustains visual function in the DBA/2J mouse model [2]. In one study, 93% of treated eyes exhibited no optic nerve damage [2], and when NAM was combined with the Wallerian degeneration slow (*Wld^S^*) transgene, neuroprotection was observed in 94% of eyes [25]. However, the substantial doses required to achieve robust protection raise concerns about clinical translatability [23]. In this context, systemic administration of nicotinamide riboside (NR) has emerged as a complementary approach, shown to elevate retinal NAD^+^ concentrations and mitigate RGC loss across in both acutely induced and chronically progressive models of optic nerve damage [23].

In addition to systemic delivery, alternative strategies have explored targeted NAD^+^ restoration at the retinal level. For example, nicotinamide encapsulated within extracellular vesicles (EV-NAM) has demonstrated enhanced preservation of dendritic architecture in RGCs, suggesting that localized delivery may confer neuroprotective benefits through early structural stabilization [27]. These findings reinforce the concept that NAM supplementation, whether systemic or targeted, can mitigate glaucomatous degeneration by preventing early morphological and functional decline of RGCs.

Recent studies have shown that, beyond its role in NAD^+^ biosynthesis, NAM may exert protective effects through the inhibition of PARP-1, a major NAD^+^-consuming enzyme activated under oxidative stress [28]. PARP-1 overactivation depletes intracellular NAD^+^ pools and suppresses SIRT1 activity, compromising cellular energy metabolism [28]. Pharmacologic or genetic suppression of PARP-1 restores NAD^+^ availability and enhances mitochondrial resilience [28].

Furthermore, its dual capacity to act as a NAD^+^ precursor and a PARP1 inhibitor may contribute to energy preservation under oxidative challenge [28,29]. While the doses used in murine studies may not be directly translatable to humans, these results form a compelling foundation for dose-finding studies in clinical trials.

### 3.3. Dendritic Preservation as an Early Neuroprotective Target

In addition to preserving somas and axons, dendritic architecture in RGCs has emerged as a particularly sensitive and modifiable target in glaucomatous neurodegeneration [30,31]. As previously described, NAM preserves dendritic complexity in a dose- and timing-dependent manner, especially when administered before the onset of IOP elevation [30]. Importantly, dendritic preservation may serve as a critical marker of early RGC stress, reflecting cellular vulnerability before irreversible somal or axonal loss occurs [30,31].

Synapse elimination is one of the earliest detectable events in RGC degeneration and correlates with functional decline in experimental models [25]. Since synaptic structures are predominantly localized on dendrites, the two are anatomically and functionally interdependent [32]. Synapse elimination often coincides with structural alterations in the dendritic compartment [32]. Therefore, dendritic degeneration may be regarded as a direct extension of synaptic pathology and serve as a morphological hallmark of early RGC dysfunction [25,32]. Mechanistically, NAM may exert this protective effect by stabilizing mitochondria within the dendritic compartment, thereby preserving metabolic capacity during early stress responses [22,25].

While EV-NAM has been shown to improve dendritic integrity in ex vivo retinal explants, its translational applicability remains uncertain due to the lack of in vivo validation [27]. Nonetheless, such targeted delivery systems represent a promising direction for enhancing the ocular bioavailability and precision targeting of NAD^+^-modulating compounds.

### 3.4. Broad Neuroprotection Across Injury Models

NAM has demonstrated broad neuroprotective effects across multiple models of glaucoma, preserving RGC somas, axons, and dendrites while reducing metabolic stress markers via stabilization of mitochondrial function and maintenance of NAD^+^ levels [25]. However, recent second-harmonic generation imaging revealed a dissociation between axonal morphology and microtubule preservation: NAM significantly preserved the overall volume of the retinal nerve fiber bundles but did not rescue axonal microtubule density [33]. This “microtubule deficit” suggests that structural preservation may not fully correspond to cytoskeletal integrity. While these findings confirm NAM’s structural protective role, they also underscore the importance of exploring complementary mechanisms to ensure cytoskeletal integrity and functional preservation.

While not a form of vitamin B3 supplementation, compounds that activate NAD^+^-dependent enzymes such as SIRT1 offer mechanistic insights into downstream pathways linked to NAD^+^ metabolism. Supporting this, a study showed that SIRT1 activation, via overexpression or resveratrol, protects RGCs from optic nerve injury by reducing oxidative stress [34]. Neuroprotection was absent in SIRT1 knockout mice, confirming its essential role [34]. Unlike NAD^+^, SIRT1 is a direct therapeutic target, offering a more precise and feasible strategy through small-molecule activation [34].

In addition to pharmacological strategies, the use of the *Wld^S^* mutation for genetic intervention has demonstrated strong neuroprotective efficacy in experimental glaucoma models [25,35]. In DBA/2J mice, *Wld^S^* expression significantly delayed RGC axon degeneration and improved functional outcomes, confirming that enhancing intrinsic axon survival mechanisms can modulate glaucoma progression [25,35]. The combined evidence underscores the varied therapeutic benefits of targeting NAD^+^-linked pathways and axonal protection mechanisms in treating glaucoma. [25,35].

These findings suggest that, beyond sustaining NAD^+^ levels, modulating NAD^+^-dependent enzymatic activity may represent an additional axis of therapeutic interest in protecting RGCs.

Collectively, these experimental findings establish NAM as a promising neuroprotective agent for glaucoma, capable of preserving the structural and functional integrity of RGCs. The identification of pre-degenerative states underscores a critical therapeutic window before irreversible neuronal damage, highlighting the need to optimize treatment timing and dosing strategies in future clinical studies. Meanwhile, targeted delivery approaches—such as EV–based systems—show potential to enhance retinal bioavailability while minimizing systemic exposure.

## 4. Clinical Evidence: Research on NAM Therapy in Human Glaucoma Management

### 4.1. Early Clinical Interventions Demonstrate Functional Improvements

Contemporary randomized controlled clinical investigations have established compelling evidence that NAM can enhance visual functionality in glaucoma patients, particularly when IOP has been stabilized through conventional medical interventions [4,36]. These findings challenge the traditional IOP-centric treatment paradigm and open the door to neuroprotective metabolic interventions.

Both trials enrolled POAG patients with medically controlled IOP who received NAM, which improved mitochondrial function and NAD^+^ metabolism, enhanced RGC energy status, and slowed functional decline independent of pressure changes [4,36]. NAM, given alone or with pyruvate, was well tolerated at high doses, with only mild gastrointestinal discomfort and no serious adverse events, and both studies called for long-term trials to confirm disease-modifying effects [4,36].

However, our analysis also revealed distinct differences in their design and outcomes. Hui et al. (2020) [4] employed NAM monotherapy, focusing on evaluating inner retinal function with electroretinogram (ERG) photopic negative (PhNR) parameters as the primary endpoint; De Moraes et al. (2022) [36] combined NAM with pyruvate, primarily measuring visual field improvement points, reflecting their emphasis on metabolic support. In terms of trial design, Hui et al. (2020) [4] conducted a longer-term crossover trial, while De Moraes et al. (2022) [36] adopted a short-term observational waiting-list design for rapid efficacy assessment. Neither study observed changes in retinal nerve fiber layer (RNFL) structure, though De Moraes et al. (2022) [36] reported a trend toward improvement. Functionally, Hui et al. (2020) [4] found improvements in PhNR and mean deviation (MD), while De Moraes et al. (2022) [36] observed significant progress in standard automated perimetry (SAP) test points, particularly in regions with early visual field loss, suggesting potential reversibility. Table 1 summarizes two representative clinical studies investigating NAM supplementation in glaucoma patients.

To better understand NAM’s therapeutic mechanisms and optimize treatment strategies, researchers have turned their attention to identifying novel non-IOP biomarkers.

### 4.2. Novel Biomarkers Beyond IOP in Glaucoma

Ongoing progress in glaucoma research has shifted focus toward mechanisms beyond IOP, recognizing glaucoma as a neurodegenerative disorder involving both metabolic and vascular dysfunctions [37]. Two recent investigations, Gustavsson et al. (2023) [38] and Petriti et al. (2024) [39], have approached this challenge from distinct but complementary perspectives, examining retinal vascular perfusion status and systemic mitochondrial function, respectively, thereby proposing novel biomarkers that may serve as valuable tools for early disease detection and individualized treatment monitoring. Although these studies focus on different biological domains, both investigations converge on a fundamental concept: glaucoma represents a multifactorial neurodegenerative disease whose progression may be driven by metabolic dysregulation and microcirculatory dysfunction.

Building on these findings, the two studies further identified promising non-IOP biomarkers for glaucoma progression by examining retinal microcirculatory perfusion and systemic mitochondrial function, potentially establishing new paradigms for disease monitoring and treatment response assessment [38,39]. These studies demonstrate that retinal perfusion density (PD) and flow indices deteriorate early in the disease course, showing positive correlations with visual field mean deviation (MD) and RNFL thickness. At the same time, glaucoma patients exhibit significantly reduced OCR in PBMCs, which closely correlates with NAD^+^ levels. These biomarker panels reflect abnormal systemic metabolic function and local vascular perfusion in glaucoma, both of which are associated with disease progression rates. Notably, OCR variability accounts for approximately 13% of visual field deterioration variance, while reduced PD predicts accelerated RNFL thinning [39].

At the vascular level, improvements in PD and flow indices correspond to optimized retinal circulation, potentially preceding detectable visual field or structural changes [38]. From a metabolic perspective, NAD^+^ level enhancement can improve PBMC OCR, suggesting the potential for mitochondrial function restoration [39]. These findings collectively indicate that PD, OCR and NAD^+^ may serve as clinically applicable composite biomarkers for quantifying glaucoma progression and treatment response.

Optical Coherence Tomography Angiography (OCTA)-derived PD changes may precede functional and structural alterations, while NAD^+^ measurements and PBMC OCR offer systemic and non-invasive advantages, establishing both as important foundations for future individualized glaucoma management and predictive model development.

### 4.3. Population-Level Evidence from the U.S. and Korea

Beyond individual clinical and mechanistic studies, nutritional epidemiology offers additional support for the role of niacin in glaucoma prevention. Five large-scale observational studies from the United States and South Korea have consistently shown that individuals consuming greater amounts of dietary niacin, a variant of vitamin B3, tend to have a lower likelihood of developing glaucoma [40,41,42,43,44]. Jung et al. (2018) [40] were the first to report in an Asian population that higher dietary niacin intake was associated with a lower prevalence of glaucoma, independent of IOP, which is particularly relevant for patients with normal-tension glaucoma (NTG). Lee et al. (2020) [41] expanded on this observation, showing that among non-obese women, those with glaucoma consumed significantly less niacin than non-glaucomatous controls. In the same study, women with a moderate body mass index (BMI, 18.5–22.9) who had POAG also demonstrated significantly lower niacin intake, suggesting that dietary insufficiency may contribute to disease risk. More recently, Lee et al. (2023) [42], using data from the 2005–2008 National Health and Nutrition Examination Survey (NHANES), reported that for every additional milligram of niacin consumed per day, the odds of developing glaucoma decreased by approximately 6%, with the protective trend being more prominent in female participants. This relationship may be mediated by niacin’s neuroprotective and vasoprotective effects, such as enhancing brain-derived neurotrophic factor (BDNF) expression and improving endothelial function [42]. Furthermore, Taechameekietichai et al. (2021) [43] found that higher-niacin consumers—specifically those within the top 50% intake groups—had significantly lower rates of glaucoma. Hou et al. (2024) [44] expanded the analysis to include multiple B vitamins and confirmed that niacin remained the most consistently protective nutrient among individuals diagnosed with glaucoma.

Although all of the aforementioned studies employed a cross-sectional design and therefore cannot establish causality, these studies consistently demonstrated an inverse association between niacin intake and glaucoma across different populations, diagnostic methods, and subgroup analyses. These findings also highlighted several potential effect modifiers, including sex, body composition, diagnostic methods, and intake range. Collectively, they support the feasibility of niacin as a potential nutritional intervention target.

In contrast, a study of Korean adults aged 60 years and older reported the opposite trend. The glaucoma group had a significantly higher mean niacin intake (12.3 ± 0.6 mg) compared to the non-glaucoma group (11.0 ± 0.1 mg, *p* = 0.046). No significant difference was observed in the nutrient adequacy ratio (NAR) between the two groups (*p* = 0.180) [45]. These findings underscore the complexity of the relationship between niacin intake and glaucoma and highlight the need for further longitudinal and interventional studies to clarify this association.

### 4.4. Clinical Safety and Tolerability Across Human Trials

Across all human NAM trials, safety profiles have been favorable [4,36,38]. In Hui et al. (2020) [4] and De Moraes et al. (2022) [36], oral NAM up to 3.0 g daily was well tolerated over 6–12 weeks with only mild gastrointestinal discomfort reported and no severe adverse events or hepatic enzyme elevations. In Gustavsson et al. (2023) [38], mild gastrointestinal discomfort was again the most frequent side effect, occurring in 37% of glaucoma patients and 33% of controls. Three participants discontinued treatment. One withdrew because of dizziness and photophobia, which were likely attributable to prolonged pupillary dilation, while the other two discontinued due to gastrointestinal discomfort [38]. Overall, NAM was well tolerated, with gastrointestinal reactions as the main limitation.

Given its favorable tolerability, oral bioavailability, and low cost, NAM represents a highly feasible adjunct to conventional glaucoma therapies [4,36]. However, caution is warranted in populations with pre-existing liver disease or concurrent hepatotoxic medications, and future long-term studies are essential for assessing chronic use risks [4,38,46].

## 5. Challenges, Limitations and Future Directions

Despite growing evidence from experimental and early clinical studies supporting the neuroprotective potential of NAM, its clinical application in glaucoma remains constrained by several critical challenges [4,27,38,39]. These challenges highlight broader shortcomings in advancing innovative therapies [3,4,13,47]. A systematic effort to overcome these barriers is essential to realize the translational potential of NAM in clinical practice.

### 5.1. Pharmacokinetics and Dose Optimization

Successful clinical translation of the aforementioned early intervention strategy remains hindered by unresolved challenges in pharmacokinetics and dose optimization, as the optimal dosing strategy for NAM in glaucoma has yet to be determined. Animal studies require doses far exceeding the commonly used 3 g in humans, raising concerns about feasibility and tolerability in clinical settings [30,47]. Although early studies have shown that daily intake of 3 g of NAM is well tolerated in the short term, evidence regarding its long-term safety remains limited [4,48,49,50].

NAM’s absorption and distribution also present challenges. Its intestinal uptake reaches a saturation point, and substantial loss occurs during first-pass hepatic metabolism and systemic methylation, limiting effective central nervous system (CNS) penetration [51]. Notably, the Nicotinamide as an Early AD Treatment (NEAT) trial—though designed for Alzheimer’s disease—employed the same 3 g NAM dose used in glaucoma research, offering pharmacokinetic insight into high-dose NAM in humans [4,36,51]. The study demonstrated that despite elevated plasma levels, systemic metabolism markedly reduced the availability of active NAM, underscoring difficulties in sustaining therapeutic concentrations at target tissues [51]. In response, researchers are investigating alternative NAD^+^ precursors such as NR, which demonstrate superior oral absorption, although their CNS bioavailability remains to be validated through further clinical investigation [23,52].

### 5.2. Combination Therapies and Synergistic Approaches

As our understanding of glaucoma deepens, it has become clear that monotherapy often falls short in addressing the complex metabolic and molecular stress driving RGC degeneration. In this context, integrated combination therapies—beyond individualized dosing strategies—are emerging as a more effective approach to enhancing the neuroprotective potential of NAM [25,26,36,53].

In animal models, similar findings have been reported. When NAM was combined with *Wld^S^*, a mimic of the cytoplasmic enzyme NMNAT2, 94% of treated eyes exhibited no glaucomatous changes—far exceeding the efficacy of either agent alone [25,26].

Clinically, De Moraes et al. (2022) [36] demonstrated that combining NAM with pyruvate significantly improved visual function compared to NAM alone. This synergistic effect likely arises from complementary metabolic support in distinct cellular compartments—NAM replenishing NAD^+^ in the nucleus and cytosol, while pyruvate sustains mitochondrial ATP production.

### 5.3. Current Large-Scale Clinical Trials Investigating Nicotinamide in Glaucoma

At present, no large-scale, multi-institutional, placebo-controlled trials of sufficient duration have been conducted to determine whether NAM can truly alter the natural course of glaucoma. Ongoing or actively recruiting clinical trials include NCT05275738 (TGNT [54]) is the largest randomized, quadruple-masked study to date, enrolling 660 participants to assess whether NAM can slow visual field deterioration over a two-year period. NCT05405868 (NAMinG [55]) (496 participants) focuses on newly diagnosed patients, assessing whether NAM can reduce visual field deterioration over 27 months. NCT05695027 [56], conducted in the United States, investigates the protective effects of combined NAM and pyruvate supplementation on visual fields and retinal structure. NCT06078605 [57], based in South Korea, evaluates short-term functional changes using ERG-derived PhNR responses. NCT06991712 [58], in Hong Kong, compares four NAD^+^ precursors for short-term visual improvement and metabolic changes. Collectively, these trials span functional and structural outcomes, highlighting the multi-faceted exploration of NAM and NAD^+^ precursors as adjunctive therapies for glaucoma. Table 2 provides a concise summary of these ongoing clinical trials.

## 6. Conclusions

NAM shows consistent neuroprotective potential in glaucoma by addressing the metabolic vulnerability of RGCs through NAD^+^ replenishment and enzymatic regulation. While promising, clinical translation remains limited by challenges in dosing, delivery, and long-term efficacy. The evolving view of glaucoma as a metabolic neurodegeneration supports a shift toward personalized, biomarker-driven treatment strategies. Dynamic indicators such as systemic NAD^+^ levels, PBMC respiration, and OCTA perfusion may guide patient selection and treatment monitoring. Combination therapies targeting multiple metabolic pathways appear more effective than monotherapy and warrant further study. Ultimately, NAM represents both a novel therapeutic approach and marker of changing paradigms in glaucoma care.

## Figures and Tables

**Table 1:** Summary of Two Clinical Trials Investigating Nicotinamide-Based Therapies in Glaucoma.

Study	Intervention	Theoretical Basis	Primary Outcome Measure	Study Design	Trial Phase	Participants	Dosage Regimen	Functional Improvement	Structural Change (RNFL ^1^)
Hui et al. (2020) [4]	NAM ^2^ monotherapy	Based on NAD^+ 3^ supplementation improving mitochondrial function and neuroprotection	PhNR ^4^ amplitude (Vmax ^5^ and Vmax ratio) via ERG ^6^	Double-masked, randomized crossover design with two 6-week periods; no washout	Not stated	57	NAM: 1.5 g/day → 3.0 g/day	Significant PhNR improvements (14.8%, *p* = 0.02); 27% showed MD ^7^ improvement ≥1 dB ^8^	No significant change
De Moraes et al. (2022) [36]	Combination of NAM and pyruvate	NAM and pyruvate combination enhances metabolic support and antioxidant defense	Number of SAP ^9^ test points improving beyond normal variability	Double-masked, randomized, wait-and-see design over ~2.2 months	Phase 2	42	NAM: 1–3 g/day + Pyruvate: 1.5–3 g/day	Median of 15 improving SAP points (vs. 7 in placebo, *p* = 0.005); mostly in mild-to-moderate regions	No statistically significant change; trend toward improvement

^1^ RNFL, retinal nerve fiber layer, ^2^ NAM, nicotinamide, ^3^ NAD^+^, nicotinamide adenine dinucleotide^+^, ^4^ PhNR, photopic negative response, ^5^ Vmax, saturated PhNR amplitude, ^6^ ERG, Electroretinogram, ^7^ MD, mean deviation, ^8^ dB, decibel, ^9^ SAP, standard automated perimetry.

**Table 2:** Ongoing Clinical Trials of NAM/NAD^+^ Precursors in Glaucoma.

Trial	Phase	Enrollment (Estimated)	Study Design	Intervention	Primary Outcome	Location
NCT05275738 (TGNT) [54]	NA ^1^	660	Randomized, Parallel, Quadruple Masking	NAM ^2^ vs. Placebo	Visual field progression (2 years)	Sweden
NCT05405868 (NAMinG) [55]	Phase 3	496	Randomized, Parallel, Triple Masking	NAM vs. Placebo	Change in visual field MD ^3^ (27 months)	United Kingdom
NCT05695027 [56]	Phase 2/3	~250	Randomized, Parallel, Single Masking	NAM + Pyruvate vs. Placebo	Visual field and OCT ^4^ structural changes (87 weeks)	United States
NCT06078605 [57]	NA	80	Randomized, Crossover, Double Masking	NAM (Mitovita) vs. Placebo	ERG ^5^ PhNR ^6^_min change (12 weeks)	South Korea
NCT06991712 [58]	Phase 2	138	Randomized, Parallel, Quadruple Masking	NR ^7^, NAM, NMN ^8^, NA ^9^ vs. Placebo	Visual field sensitivity (2 weeks), plasma NAD^+ 10^ metabolites	Hong Kong

^1^ NA, not applicable, ^2^ NAM, nicotinamide, ^3^ MD, mean deviation, ^4^ OCT, optical coherence tomography, ^5^ ERG, electroretinogram, ^6^ PhNR, photopic negative response, ^7^ NR, nicotinamide riboside, ^8^ NMN, nicotinamide mononucleotide, ^9^ NA, nicotinic acid, ^10^ NAD^+^, nicotinamide adenine dinucleotide^+^.

## Data Availability

No new data were created or analyzed in this study. Data sharing is not applicable to this article.
